# Living with Aliens: Effects of Invasive Shrub Honeysuckles on Avian Nesting

**DOI:** 10.1371/journal.pone.0107120

**Published:** 2014-09-17

**Authors:** Jason M. Gleditsch, Tomás A. Carlo

**Affiliations:** Department of Biology and Intercollege Graduate Degree in Ecology, The Pennsylvania State University, University Park, Pennsylvania, United States of America; University of Marburg, Germany

## Abstract

Invasive species have come to the forefront of conservation biology as a major threat to native biodiversity. Habitats dominated by shrub honeysuckles (*Lonicera* spp.) in the United States have been characterized as “ecological traps” by ecologists. Here we tested this hypothesis by investigating the effects of shrub honeysuckles on the nesting ecology of native birds in seven study sites in central Pennsylvania, USA. We examined how the abundance of shrub honeysuckles influenced the selection of nesting substrates and habitat for a community of common songbirds, and the parental-care behavior and nestling development of gray catbirds (*Dumetella carolinensis*). We found that birds had a strong bias towards nesting in honeysuckle shrubs, but not necessarily for nesting in honeysuckle-dominated habitats. Nest predation rates were affected by the density of nests in a habitat, but not by the overall abundance of honeysuckles in such habitats. Honeysuckle abundance in the habitat did show significant effects on some parental-care behavioral parameters: catbirds had higher nest visitation rates and shorter visit lengths in areas of high honeysuckle density. On average, Gray catbirds fed fruit 12%±0.31 s.e. of their nestling-feeding bouts, mostly fruits of shrub honeysuckles. Nestlings in sites with high honeysuckle density also showed higher mass:tarsus ratios, suggesting a good (possibly better) physiological condition of catbird nestlings at the time of fledging. Our study shows that honeysuckle-dominated habitats could have equivocal effects on nesting parameters of common species of native birds. We advise more caution in the widespread denomination of novel plant communities with high densities of honeysuckle as “ecological traps” as effects can be null or positive on native birds in certain localities.

## Introduction

The ecological interaction between invasive plant species and bird communities has received a fair amount of attention over the past fifteen years [1]. It is increasingly recognized that the effects of these relationships are often species specific, context-dependent, and often involving mixes of positive and negative effects [1]–[4]. Thus, it is critical to better understand the outcomes and complexities of invasive plant-native bird interactions to create and implement effective conservation practices on landscapes with a long history of human disturbance and novel mixes of introduced and native species [1], [3]. Invasive species of bird-dispersed fruiting shrubs, such as honeysuckles (*Lonicera* spp.), now serve as foraging and nesting substrate for many species of native songbirds [4]–[6]. In fact, at scales of landscapes or forest patches, the diversity and abundance of some native bird species appear positively correlated with the abundance of shrub honeysuckles, which now provide the most abundant source of fruit resources in the summer and fall for vast regions of the Midwest and Eastern North America [4], [7]. However, the ecological effects of shrub honeysuckles are largely viewed as detrimental despite widespread use – and effective dispersal – by many native songbird species [8]–[9].

The effect of shrub honeysuckles on nest predation rates has been a major focus in the literature. For example, studies have shown that northern cardinals (*Cardinalis cardinalis*) and American robins (*Turdus migratorious*) have higher nest predation rates nesting in honeysuckle than nesting in native shrub species [8], [10]. It is the combination of higher nest predation with the tendency for birds to favor honeysuckle as a nesting substrate that has led ecologists to label honeysuckle as an ecological trap. However, nest predation is only one way in which honeysuckle can affect bird populations. For example, bird populations are assumed to be negatively affected by honeysuckle because introduced plants support less biomass and diversity of foliar arthropods [11]. Further, honeysuckle fruits have been described as having poor nutritional quality [12], leading researchers to speculate that they can lead to the malnutrition of bird consumers [5].

Invasive shrub honeysuckles can alter avian ecology in several ways. First, by fruiting earlier in the spring than most native fruiting species, they could impact the development and survival of nestlings by changing the types, amount, and quality of resources available for nestling care during the breeding season, particularly for those species that rely heavily on fruit [13]. On the other hand, it can be hypothesized that habitats dominated by shrub honeysuckles could provide opportunities for the caring and feeding of nestlings as they could improve foraging and feeding activities for frugivorous birds, given the typically high fruit abundance of such habitats [4]. But the low fruit quality of honeysuckles [5] suggests that it could reduce parental physiological condition and hinder the development of nestlings. This reasoning suggests that increasing the amount of fruit in nestling diets in such habitats would have negative impacts on their development.

Here we investigated the use of nesting habitat and nest substrates of birds at seven sites in central Pennsylvania, U.S.A to test several aspects of the ecological trap hypothesis. Each site contained areas of low, medium, and high abundance of shrub honeysuckles. Based on the ecological trap hypothesis, first we predicted that nest densities would be greater at higher abundances of honeysuckle in the habitat (prediction 1), and that birds will disproportionately build nests in honeysuckle shrubs as compared to other available shrubs (prediction 2). Next, we examined the effects of honeysuckle-dominated habitats on aspects of the parental care behavior and nestling development of gray catbirds (*Dumetella carolinensis*). For this we predicted that catbirds would show different parental-care behavioral patterns in honeysuckle dominated habitats than native habitats (prediction 3). We also hypothesized that nesting in honeysuckle dominated habitats would negatively affect the development of nestlings (prediction 4). Last, we report on nest predation rates as affected by nest placement in honeysuckle or native shrub substrates, and as a function of the amount of shrub honeysuckles in the habitats.

## Methods

### Ethics Statement

This study was carried out in accordance with the recommendations of the Institutional Animal Care and Use Committee (IACUC) of the Pennsylvania State University. The protocol (including the portion that involved handling birds) was approved by the IACUC of the Pennsylvania State University (Permit Number: 37069), Pennsylvania State Game Commission (Permit Number: 77-2011), and by the U.S. Fish and Wildlife Service (Permit Number: MB44836A-0). No endangered species were used in this study and permission was obtained to conduct research on migratory birds, which are protected, from the U.S. Fish and Wildlife Service (Permit Number: MB44836A-0). In addition, permission was obtain from Pennsylvania State University Forestland Management and the Centre County Parks and Recreation to conduct this research on their lands.

### Study sites

We selected seven sites in central Pennsylvania, USA that varied in their amounts of honeysuckle. The sites were owned by the Pennsylvania State University or by the Centre County Parks and Recreation and were at least 1 km apart. In addition, all of the sites had an understory dominated by shrubs and a heterogeneous canopy, including gaps, and open areas with scattered trees. The sites differed primarily in the community of shrubs present. In four of the sites, honeysuckle species were dominant (>50% *L. morrowii* and/or *L. maackii*); Site 1–12.5 ha searched, Site 2–5.1 ha, Site 3–6.9 ha, and Site 4–1.7 ha). The other three sites (<50% honeysuckle; Site 5–0.4 ha, Site 6–0.4 ha, and Site 7–3.8 ha) were all classified as dominated by native shrubs (See Appendix S1 for site coordinates and characteristics). Differences in area among study sites reflects the fact that shrub habitats not dominated by honeysuckle species are extremely rare, and thus we were unable to find larger areas dominated by native shrubs.

### Vegetation cover sampling

Vegetation cover was measured using systematic surveys. Survey points were established by creating a thirty-meter grid at each site. At each point, we estimated the proportion of the area covered by all woody plants above 0.5 m tall within a two-meter radius. The total number of vegetation points per site was Site 1 (191), Site 2 (74), Site 3 (134), Site 4 (42), Site 5 (27), Site 6 (24), and Site 7 (58). The cover data was then used to create a honeysuckle density map with the inverse distance weighting (IDW) interpolation tool in ArcMap 10 (ESRI). ArcMap classified cover in three categories: high honeysuckle cover (>60%; hereafter HHS), medium honeysuckle cover (>30 and ≤60%; hereafter MHS), and low honeysuckle cover (≤30%; hereafter LHS, see Appendix S2 for more detail). We chose these categories to simplify the models and to reflect the heterogeneity of the habitat types with the low and high categories being more homogeneous in their shrub communities. The area (m^2^) of each classification in each site was then calculated. The total area of the three habitat classifications combined across all study sites was fairly even and amounted to: 12 ha for LHS, 10 ha for MHS, and 9 ha for HHS.

### Nest searches

We conducted nest searches in the spring and summer of 2011: one at the start of the breeding season (May 8^th^–21^st^) and the other when most bird species lay a second clutch (June 19^th^–July 2^nd^). On every search day, two observers visited two sites in the morning and searched for approximately four hours, and then the observers switched between the sites in the afternoon, where they searched for an additional four hours (i.e., to prevent observer-induced biases in nest detection). The whole area of each study site was searched for new nests once every week controlling for nest search effort (approximately equal hours per unit are per site), resulting in a total of 224 nest-search hours for each of the two nest search rounds (total  = 448 hrs). Multiple nest searching techniques were employed (i.e. territory searches, parental behavior following, systematic searching) to reduce the nest detectability biases across sites and habitats. Nests were mapped using a Magellan Professional sub-meter GPS with ArcPad 7 software (ESRI). We recorded the height of nests; the species of plant nests were built in; and the clutch size. The nests were then monitored through the whole breeding season by revisiting them every other day to assess their status. For predation analyses, nests were classified as either successful or predated. Successful nests fledged nestlings, while predated nests showed evidence of predation (i.e. loss of nestlings, pieces of nestlings, nest damage). Because we made detailed maps of the plant communities in each study site, our study design allowed us to examine the effects of background levels of honeysuckle both at the scale of the nest vicinity, and at the broader habitat-level scale.

### Catbird parental care behavior

To test the hypothesis that catbirds would show behavioral patterns that could increase nest predation in honeysuckle-dominated habitats, 32 catbird nests were used for the analysis of parental care behavior. The gray catbird was chosen as the focal species for this test because it has been shown to have a strong relationship with honeysuckle [4] and it was the numerically dominant species in our sites. To assess parental care parameters we made video recordings of the 32 nests using a Samsung Smart Flash Memory Camcorder (SMX-F40BN). Nests were filmed when catbird nestlings were 4–5 days old. Each video recording lasted approximately 4.5 hours in the morning, and then repeated again in the late afternoon. Cameras were set up no closer than 1 m to the nests and were housed in a non-reflective, black plastic box to reduce the potential disturbances created by the recording. For all nests and sites, cameras were placed in the morning between 07:00–08:30 and between 15:00–16:30 in the afternoon. From each video we determined the total time spent present at the nest, the number of visits, and when possible, the type of food being fed to the nestlings (fruit or invertebrate). The rationale for measuring these behavior parameters was that the risk of predation can increase with the higher rates of nest visitation and activity necessary to meet nutritional requirement of nestlings in a poor quality habitat [14]. We excluded from analyses any nests that were obscured from view in the sampled video or had fewer than three nestlings in the nest (all nests had 3–4 nestlings), resulting in a total of 23 recordings suitable for the analysis of parental behavior (8 nests in Site 1, 2 in Site 3, 4 in Site 7, 2 in Site 2, 3 in Site 6, 4 in Site 5). Of these, twelve nests were in honeysuckle-dominated habitat (HSD) and eleven in habitat dominated native shrub species (NAT). To classify the habitat of recorded nests we averaged the cover types from vegetation points found within a 25 m radius from the sampled nest Nests found in areas with an average honeysuckle cover >50% were classified as being in honeysuckle dominated habitat (HSD; average honeysuckle  = 60.7%±3.18 s.e.), and the nests found in an average honeysuckle cover of ≤50% were classified as being in a habitat dominated by native species (NAT; average honeysuckle  = 4.3%±1.84 s.e.).

### Nestling development measurements (prediction 4)

Fourteen of the 32 nests recorded were then sampled to examine the condition of the nestlings. The other 18 of the 32 nests were unable to be sampled due to predation and early fledging. To assess nestling condition, two nestlings were randomly selected from each nest when the nestlings were approximately 8–9 days old, totaling 28 nestlings. We sampled the nestlings when they were 8–9 days old to reduce the risk of forced fledging due to handling but also to maximize the assessment of their pre-fledging condition. Body mass (g) and tarsus length (mm) were recorded for each nestling. To control for allometric effects of variation in body size, we divided the mass of the nestlings by the tarsus length [15]. We expected a lower mass to tarsus ratio in nestlings in HSD habitats than in NAT habitats caused by nutritional deficiencies that result from including a higher frequency of nutrient-poor fruits (i.e., honeysuckles) in the diet of nestlings.

### Statistical Analyses

#### Selection of habitat and nest substrate

To test the prediction (1) that honeysuckle-dominated habitats have higher densities of songbird nests (irrespectively of the nest substrate) we used a One-way ANOVA. We calculated nest density, the response variable, by dividing the number of nests found in each habitat classification within a site (i.e., Low, Medium, or High honeysuckle, see Appendix S2), by the area covered by the habitat in the site. This yielded a total of seven independent nest density estimates for each habitat classification (one from each study site) to conduct the analysis. Next, to test for the prediction (2) that nests are preferentially constructed in honeysuckle substrates we used a One-Way ANOVA. As response we used the number of nests built in two substrate classifications within each site: honeysuckle and non-honeysuckle (mostly native spp.) divided by the availability (area) of the substrate classification obtained from the vegetation cover data.

#### Catbird behavior and nestling development

To test predictions that catbirds would exhibit different parental behavior in areas of high honeysuckle abundance (3), we used one sided *t*-tests to compare the average nest visitation rates of parents (visits per minute) and the average length of visits (minutes per visit) between HSD and NAT habitats. We used generalized linear models (GLMs) with binomial error distributions to compare nest visitation rates, as well as the proportion of visits in which parents fed fruit to nestlings, in HAS and Nat habitats. We addition, we used a one-sided *t*-test to compare the average mass to tarsus ratio of nestlings found in NAT habitats to those in HSD habitats to test our prediction that nestlings in HSD had reduced development (4).

#### Nest predation at habitat and substrate scales

To examine whether nest predation rates are higher in honeysuckle-dominated habitats we used a General Linear Mixed Model with binomial error distribution. As response variable we used both the number of predated nests and the total number of nests per habitat classification (again, Low, Medium, or High honeysuckle) per site. We included nest density per habitat in each site as a covariate since density is expected to influence predation rates [16]. To examine if honeysuckle substrates increase nest predation rates compared to native shrub substrates we also used the General Linear Mixed Model with binomial error distribution. As response we used both the number of predated nests and the total number of nests per substrate classification per site (at two levels: honeysuckle and non-honeysuckle substrates) and the density of nests in each site as a covariate. We point out that results from these analyses should be interpreted some with caution because we were not able to account for the effect of exposure time on predation rates.

## Results

**Figure 1:**
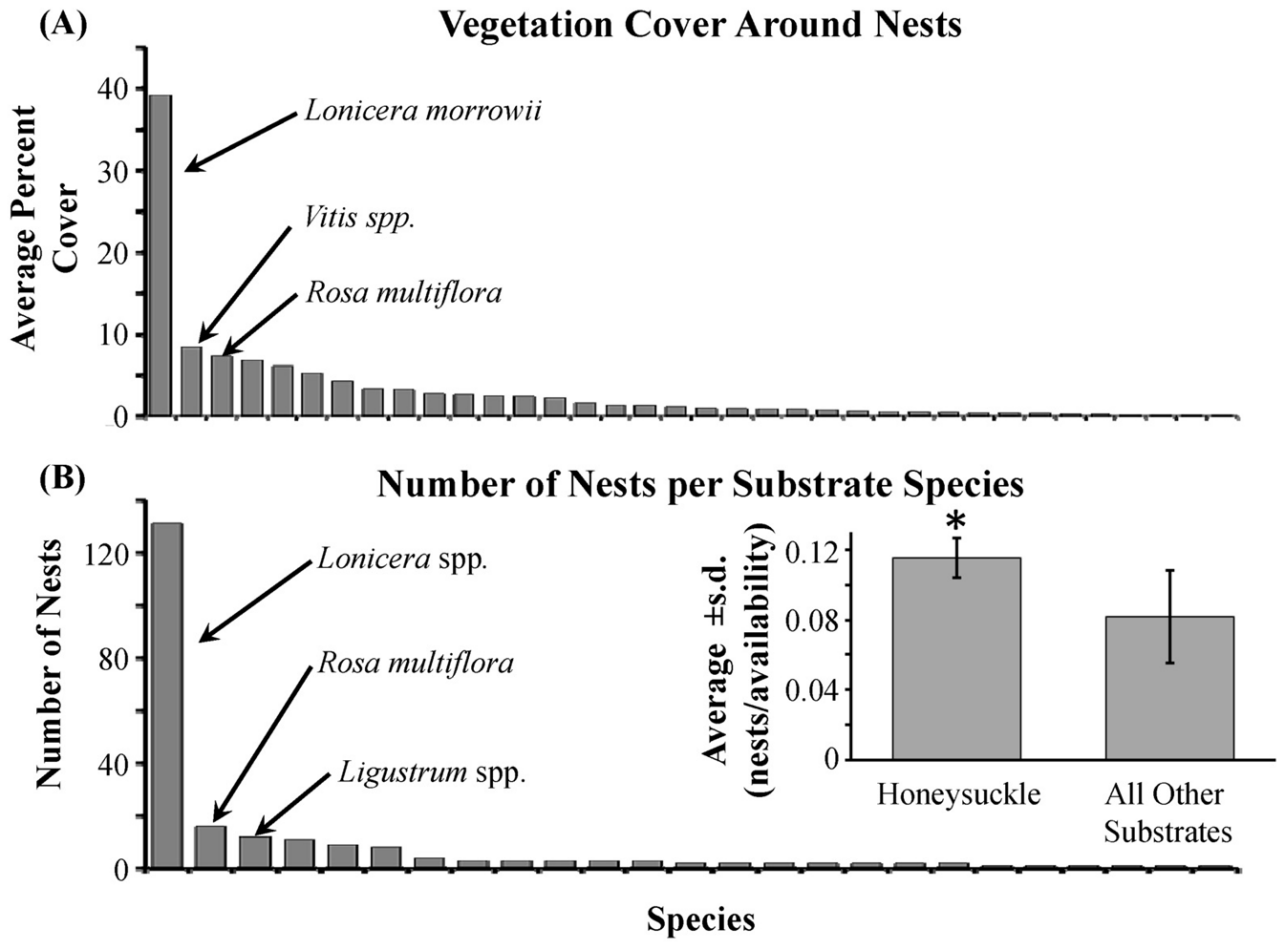
Honeysuckle species (*Lonicera maackii* and *L*. *morrowii*) were the dominant vegetation cover around the nests and was also the most used nesting substrate. Panel A shows the average cover of each plant species within a 2 m radius around nests found in the study sites. Morrow's honeysuckle (*L*. *morrowii*) was the dominant species found in the vegetation cover analysis. Panel B shows the number of nests built in each substrate (i.e., plant species). More nests were built in honeysuckle species than any other shrub used for nesting substrate. The inset shows the average number of nests found in each substrate category (i.e., honeysuckle spp., and all other plant species). We found significantly more nests built in honeysuckle species than in all the other species given the availability of each substrate category. (*P≤0.05).

We found 227 nests of 10 bird species (Appendix S3). Number of nests found were distributed across sites as follows: Site 1 = 102, Site 2 = 26, Site 3 = 36, Site 4 = 13, Site 5 = 12, Site 6 = 12, Site 7 = 21 (Appendix S4). The three numerically dominant bird species found nesting in the sites (gray catbird, American robin, and northern cardinal; Appendix S3) have similar nesting ecology (i.e. nest location, nestling care, nestling feeding habits). The nests found were built in 25 plant species including trees, shrubs, and grasses. Of the nests, 58% were built in honeysuckle (Figure 1). The plant communities observed around the nests consisted of at least 39 plant species including both native and exotic plant species (Figure 1A; Appendix S5). The most abundant plants in the cover around the nests and used as nesting substrate were shrub honeysuckles with an average percent cover of 38.41%±2.9 s.e. (Figure 1B and inset). The parameter of Site was tested in all analyses and was found to be insignificant in all cases.

### Selection of habitat and nest substrate

**Figure 2:**
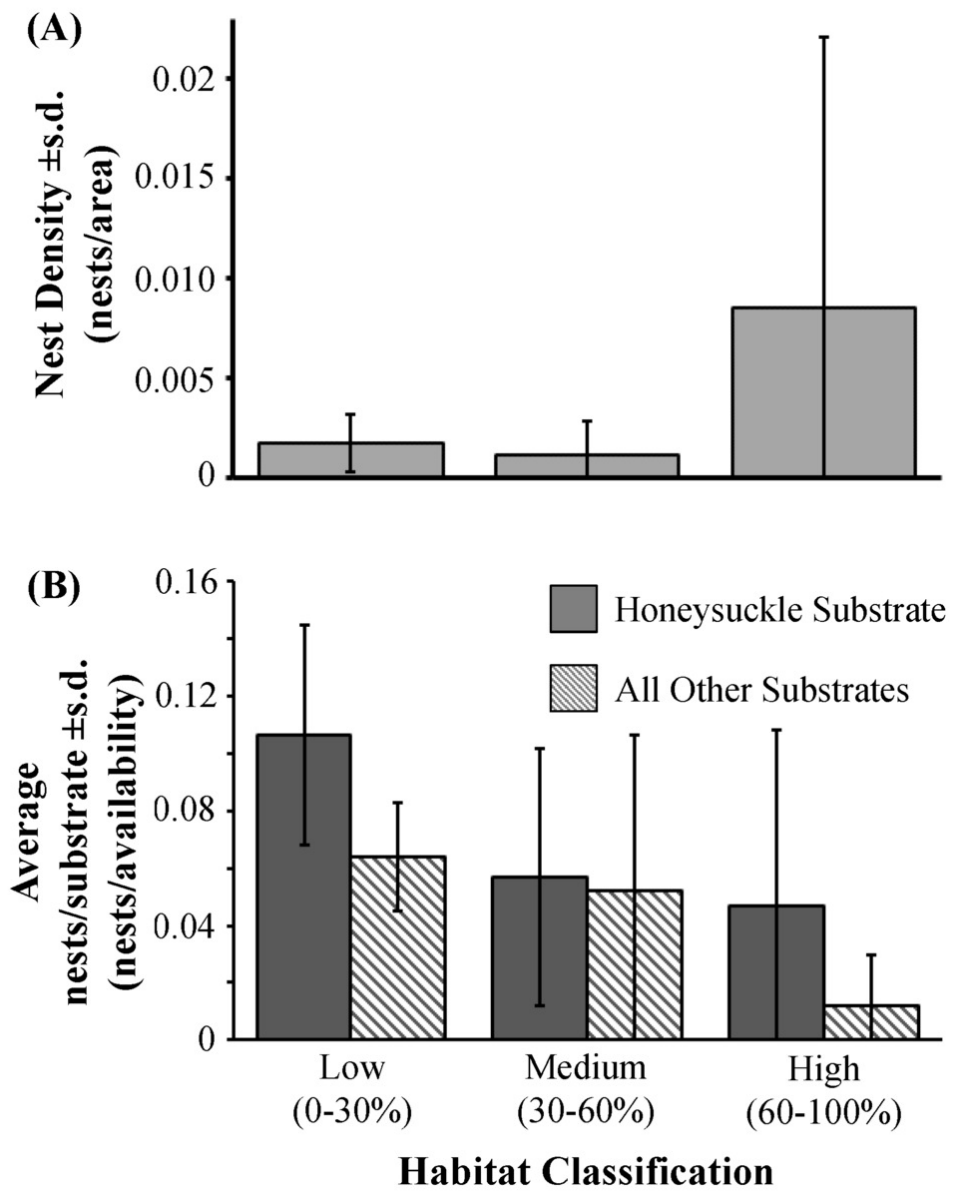
Birds did not show a bias for honeysuckle-dominated habitat (*Lonicera* spp.), but did show a bias for honeysuckle species as a nesting substrate. Panel A shows the average density of nests in the low (0–30% honeysuckle cover), medium (30–60%), and high (60–100%) honeysuckle habitats. We did not find a significant trend of birds to nest in any of the habitat classifications disproportionately. Panel B shows the average number of nests per substrate classification (honeysuckle spp. and all other species) in the three habitat classification. We found significantly more nests built in honeysuckle plants irrespective of how abundant were honeysuckles in the habitat.

The density of nests was not influenced by habitat classification (i.e., high, medium, and low) (Table 1; Figure 2A). However, habitat classification had a significant effect on the nest substrate usage of the shrub-nesting bird species (Table 1). The shrub-nesting birds used honeysuckle as nesting substrate significantly more frequently than other shrubs available for nesting substrate given the availability of substrates in the environment (Table 1). The bias of birds toward nesting in honeysuckle substrates was independent of habitat classifications based on honeysuckle since there was no interaction between ‘habitat’ and ‘substrate’ factors (Table 1; Figure 2B). At the local neighborhood scale, shrub-nesting birds chose honeysuckle substrate more frequently than other shrubs (Table 1; Figure 1B inset).

**Table 1:** Results of three ANOVAs testing the hypothesis that shrub nesting birds had a bias for a habitat type (Prediction 1), a bias for a substrate type given the availability of that substrate in the local nest environment (Prediction 2a), or a bias for a substrate type given the abundance of substrates per habitat type at each site (Prediction 2b).

Model	Response in model	Parameter	DF	*F* ratio	Prob.>*F*
Prediction 1	Density of Nests	Habitat	2	1.8729	0.1824
					
Prediction 2a	Number of Nests/Availability	Substrate	1	9.3414	**0.0100**
					
Prediction 2b	Number of Nests/Availability	Habitat	1	14.8572	**0.0008**
		Substrate	1	7.1347	**0.0134**
		Habitat*Substrate	1	0.0734	0.7888

Included in the table are the response variables for each ANOVA and the parameters included in each model. Prediction 1 and 2a had only one parameter in the ANOVAs and Prediction 2b had two parameters and the interaction between those parameters (model fit: *F* = 7.3551, DF = 3, *P* = 0.0012). The factor “site” was not significant in any of the full models and was thus excluded.

### Catbird behavior

**Figure 3:**
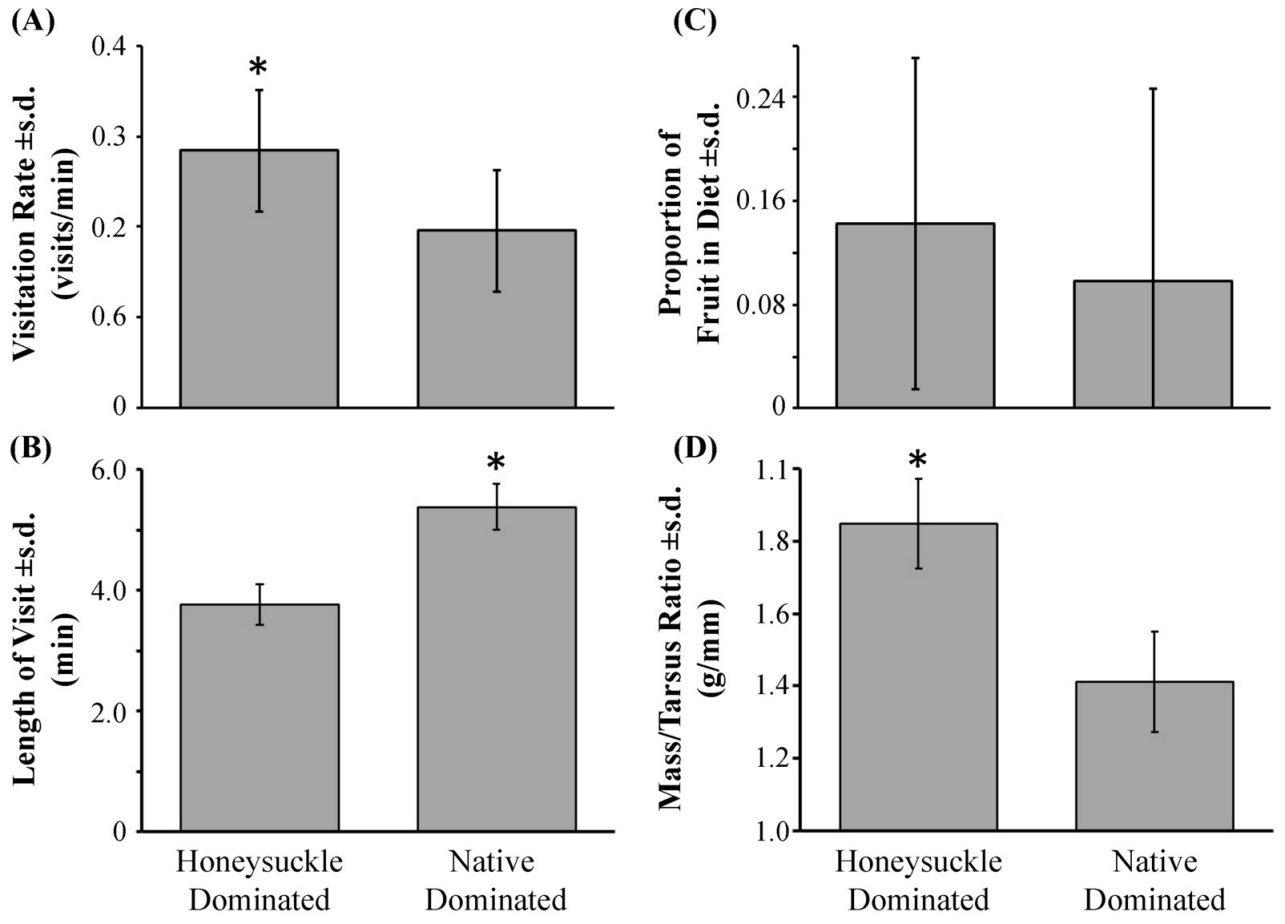
Higher visitation rates and shorter nest visit lengths were observed for catbird nests found in honeysuckle (*Lonicera* spp.) dominated habitats. In addition, nestlings were observed to be in equal or better condition at the time of fledging. Panel A shows the average visitation rate in the honeysuckle dominated (HSD) and the native dominated (ND) habitats. We found higher visitation rates in HSD habitats. Panel B shows the average length of each visit in HSD and ND habitats. We found that parents were at the nest longer in ND habitat. Panel C shows the average proportion of fruit in the diet of the nestlings in HSD and ND habitats. We did not find a significant difference between habitat types for this response. Panel D shows the average mass:tarsus ratio of nestlings in HSD and ND habitats. We found a higher mass:tarsus ratio in HSD habitats. (*P≤0.05).

For the 23 catbirds nests we collected a total of 197.5 hours of video (see Appendix S6 for raw data), revealing that nests located in HSD habitats had visitation rates 44% higher than nests located in NAT habitats (Figure 3A; *t* = 3.450, DF = 21, *P* = 0.002). However, there was no difference between HSD and NAT habitats in the proportion of feeding bouts (*z* = 1.718, DF = 21, *P* = 0.086), or in the proportion of feeding bouts in which nestlings were fed fruit (Figure 3C; *z* = 1.037, DF = 19, *P* = 0.300). There was no difference in the average total time that catbirds spent at nest in HSD or NAT habitats (*t* = 0.651, DF = 21, *P* = 0.522). However, the average duration of visits was significantly higher for nests in NAT compared to HSD habitats (Figure 3B; *t* = 2.188, DF = 21, *P* = 0.04).

### Catbird nestling development

Nine of the 14 nests sampled for nestling condition at fledging were found in the HSD habitats while five were from NAT habitats (see Appendix S7 for raw data). There was not a significant difference in the weights of the nestlings between HSD and NAT habitats (DF = 26, *t* = 0.459, *P* = 0.650). The ratio between the body mass of the nestlings and the length of their tarsus was significantly higher for birds in HSD habitats compared to NAT habitats (Figure 3D; DF = 26, *t* = 2.062, *P* = 0.049), with an average magnitude of increase of 4% (Figure 3D).

### Nest predation at the scale of habitat and substrate

**Figure 4:**
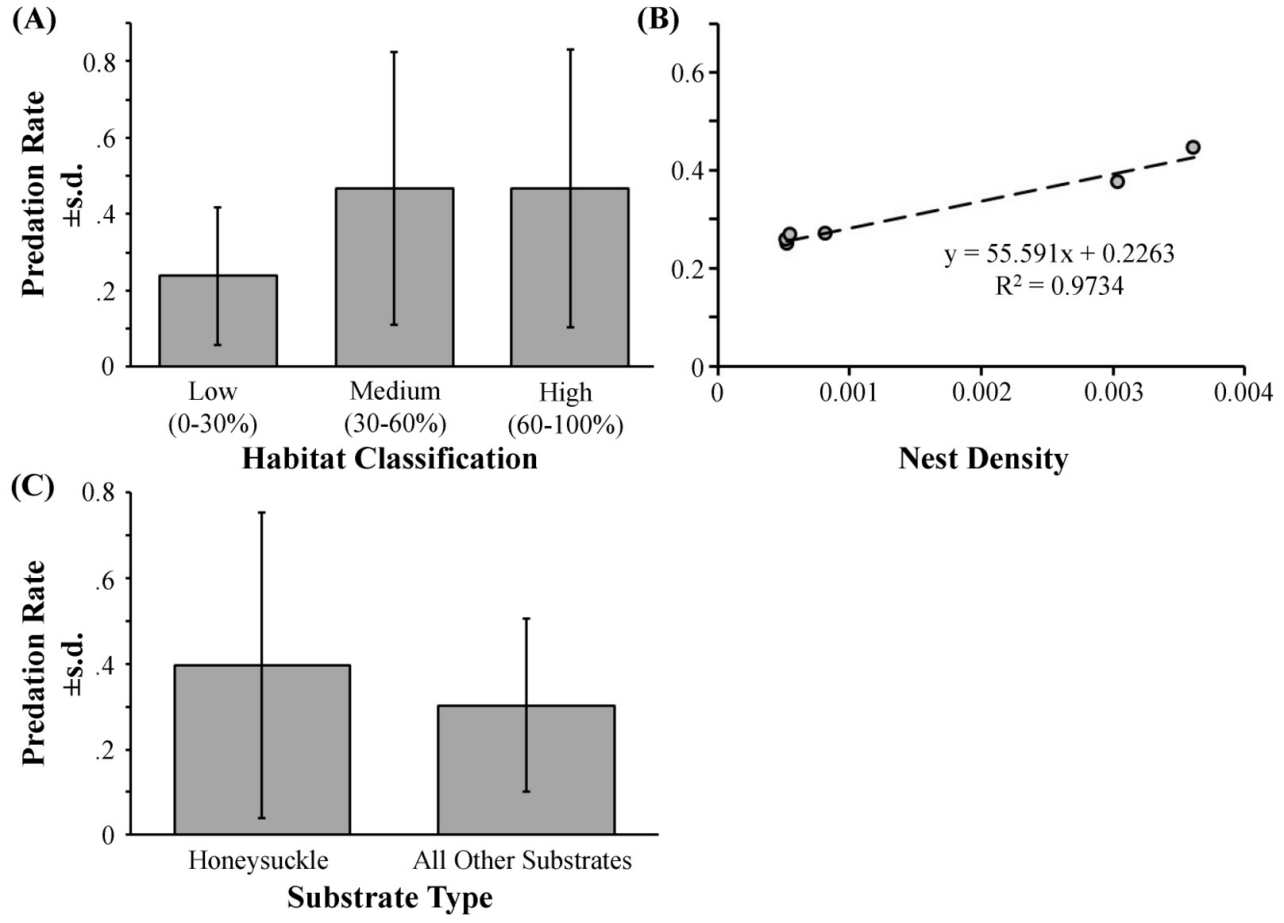
There was no relationship between honeysuckle (*Lonicera* spp.) abundance or honeysuckle use as nesting substrate and the predation rate of nests. Panel A shows the average nest predation rates in the low (0–30% honeysuckle cover), medium (30–60%), and high (60–100%) honeysuckle habitats. Panel B shows the nest predation rates of the sites regressed with the nest density of each site. We observed a strong relationship between nest density and the predation rate of nests. Panel C shows the average nest predation rates in the different substrate classifications (honeysuckle spp. and all other species).

Twenty-nine percent of the nests that we monitored during the study year failed (Appendix S4). The sites that had the highest predation rates were Site 5 (44.4% predation, NAT habitat) and Site 6 (37.5% predation, NAT habitat). Lower predation rates were observed at Site 7 (26.7% predation, NAT habitat) and Site 3 (25.8% predation, HS habitat). Neither the habitat classifications nor the nesting substrate classifications had a significant effect on nest predation rates (Figure 4). Only the density of nests in the habitat was correlated with nest predation rate (Figure 4).

## Discussion

While some predictions of the ecological trap hypothesis found support in our results, most were not supported. The ecological trap hypothesis is supported in that a focal bird species – the gray catbird – modified its nest-tending behavior in unfavorable ways when breeding in habitats of high honeysuckle abundance (HSD) as compared to nesting in habitats dominated by native shrub species (NAT). Catbirds visited nests more frequently in HSD habitats than in NAT habitats, a pattern that could increase nest predation rates in HSD habitats [14]. Our results also show, in agreement with previous studies, that shrub-nesting birds in central Pennsylvania have a positive bias for building nests in shrub honeysuckles, but this tendency is unrelated to the habitat-level abundance of honeysuckle, or to predation rates. Thus, the ecological trap hypothesis is rejected in that habitats with more honeysuckle does not necessarily have higher nest densities than areas dominated by native shrubs because habitat and substrate classifications, based on honeysuckle abundance and use, were poor predictors of nest densities across the seven study localities. Furthermore, the ecological trap hypothesis is rejected in that catbird nestlings showed signs of being of at least equal body condition in HSD habitats compared to NAT habitats as suggested by the greater mass-to-tarsus ratios found in nestlings raised in HSD habitats. To our knowledge, this is the first study to test the effects invasive shrubs on bird nesting ecology in multiple localities, and to demonstrate changes in parental care behavior in response to habitat changes related to the abundance of invasive plant species.

### Selection of habitat and nest substrate

More nests were built in honeysuckle shrubs than expected by chance across study sites, and the trend was consistent irrespectively of the abundance of honeysuckle in the plant community background. Even in habitats with very low honeysuckle abundance, such as those found in the Site 7 and Site 6, more nests were found in honeysuckle substrates, demonstrating a strong bias in favor of the introduced shrubs. Previous studies that have examined nest substrate selection in the context of honeysuckle invasions have focused only on the local nest environment to calculate measures of substrate availability (e.g., [8]), and only a handful of studies have considered scales larger than the immediate nest environment (e.g., [17]).

Curiously we did not find positive correlations between nest densities and honeysuckle abundance, which refuting the hypotheses that the early leaf phenology of honeysuckle increases nest densities [9], as has been shown for other study systems [18]. It is likely that other factors, such as competition and predation [19] (see Appendix S8 for number of predated nests), are influencing nest habitat choice more strongly in our study sites than the composition of the shrub community. Another possibility is that the composition and structure of the songbird community influenced the habitat-level selectivity of some of the species. There were differences in the composition of the bird community across our study sites (see Appendix S5), but the ways these differences impact nest densities and predation are beyond the scope of this study and remain to be addressed in the future. Understanding habitat-level selection is inherently difficult since multiple factors such as resource availability [20], competition [21], source-sink dynamics [22], demographic effects [23], and predation risk [19] can influence the presence of species in habitats. On the other hand, the bias for substrates is more straightforward to interpret. For many passerine species, substrate selection is based primarily on how well birds can conceal nests from predators. Birds are probably attracted to honeysuckle substrates for nesting because of its early leaf flush and the dense architecture of its branches.

### Catbird care and nestling development

Catbirds had a 45% higher visitation rate which means they made significantly more trips to feed their nestlings but spent 32% less time per visit, in habitats of high honeysuckle cover (HSD). There are a few ways in which these results could be interpreted. One such way is that birds spend more time per visit in habitats dominated by native vegetation because the available food resource were of higher quality, providing more energy per volume and thus reducing foraging effort. The lower volume to energy ratio of fruit compared to invertebrates [5], [24] could explain the higher visitation rates observed in HSD habitats in support of the ecological trap hypothesis, given that HSD habitats have more fruit, and fewer foliar invertebrates, than NAT habitats [4], [11], [25]. With this interpretation of our results, it could be speculated that the resulting body and developmental condition of nestlings in HSD habitats will be of lower quality. However, judging from the greater average mass-to-tarsus length ratio found in the nestlings of catbirds breeding in HSD habitats, it seems that developmental condition in such habitats is equal or better to those of nestlings raised in the studied NAT habitats [26].

Our records of honeysuckle fruit being fed to catbird nestlings in both HSD and NAT habitats is remarkable, and it suggests that honeysuckle, or fruit in general, is an important resource for both adult catbirds and their nestlings, even at low dietary percentages. Underscored is the fact that honeysuckle fruits were fed to nestlings even in sites with a very low abundance of honeysuckle cover (e.g. Site 5, Site 6, and Site7). In temperate regions, fruit densities are typically low during the nesting period of landbirds [27]. But fruit of introduced species such as shrub honeysuckles are changing the picture, offering fruit resources to breeding birds at levels that probably surpass historical fruit densities in breeding habitats at northern latitudes, at least in recent history. As an alternative to the ecological trap notion, we hypothesize that the availability of early-ripening fruits, such as those of shrub honeysuckles can help some birds achieve an equal or greater (not lesser) nutritional condition in highly disturbed habitats. Even though it has been shown that honeysuckle fruits possess high carbon to nitrogen ratios which is an indication of poor nutritional quality [5], they may also possess potentially important secondary compounds and nutrients that can be difficult to detect. Our records of catbirds feeding their nestlings with fruit in all sites, and the finding that nestlings in habitats dominated by shrub honeysuckles appear to be in good body condition (judging by their mass:tarsus length ratios) provide provisional support to this notion.

### Nest predation at habitat and substrate scales

Unlike previous studies, we did not find nest predation rates to vary significantly among the different habitat classifications that reflected the abundance of shrub honeysuckles (Figure 4). Neither did we find nest predation rates to be significantly different according to whether nests were built in honeysuckle or other substrates (Figure 4). The variable explaining increases in nest predation rates was nest density, and not any of the habitat or substrate classifications. This is an important finding because previous studies show that American robins and northern cardinals suffer more predation when nesting in shrub honeysuckles [8], [10]. Several factors may explain this discrepancy. First, in this study, we investigated many bird species rather than one, which may mask species-specific effects. Second, previous work on nest predation in habitats of high honeysuckle abundance have not accounted for the effects of the overall nest density in the habitats. Nest density is a key variable because predation rates. This is due to the common positive density-dependent responses of predators to prey density [14]. Thus, not accounting for density effects can confound “habitat” categorizations. Thus, the failure of previous studies to consider different scales and to account for the effects of nest density concurrently with substrate and habitat classifications limits their interpretation of causality.

## Conclusions

We did not observe a preference of birds to breed in habitats dominated by shrub honeysuckles across seven study sites in central Pennsylvania. We did find a preference for birds to build their nests in shrub honeysuckles in all sites, which is in agreement with what previous studies have reported. We also found that two aspects of the parental care behavior of gray catbirds (i.e., the frequency of nest visitation and the time spend per nest visit) were different in areas dominated by honeysuckles in comparison to areas dominated by native vegetation. But those differences did not seem to cause nestlings to be in lower body condition at the time of fledging in honeysuckle dominated habitats, or to suffer more predation. In fact, mixed diets have been shown to be beneficial for many birds [28], and the high abundance of honeysuckle fruit can facilitate diet mixtures.

Our results show that relationships between introduced fleshy-fruited plants and native birds are complex and not easily characterized as purely harmful or beneficial because they can include negative, neutral, or positive outcomes [1]–[3]. It is important to consider the possibility that habitats and plant communities that are novel and self-assembled, such as the shrublands that now cover much of central Pennsylvania and the American mid-west, could now be important as breeding habitat for many native bird species, and ultimately confer resilience to avian and plant communities to withstand future anthropogenic pressures and climatic changes. Our results show that categorizations such as “ecological traps” can be fallacious, ephemeral, and/or not broadly applicable, and suggest that the traditional and widespread categorical approach to invasive species management should be revised to prevent harming certain communities and ecosystems, especially areas in a process of self-recovery from heavy human disturbances.

## Supporting Information

Appendix S1 **Characteristics of the seven study sites.**
(DOCX)

Appendix S2 **Average honeysuckle cover per habitat classification for each study site.**
(DOCX)

Appendix S3 **Number of observed nests built during the study year for the bird community.**
(DOCX)

Appendix S4 **Number of observed nests built during the study year in each habitat classification and substrate type per study site.**
(DOCX)

Appendix S5 **Percent cover of plant species around nests.**
(DOCX)

Appendix S6 **The parental care data obtained from video recordings taken when the nestlings were 4–5 days old.**
(DOCX)

Appendix S7 **The morphological measurements of nestlings at age 8–9 days.**
(DOCX)

Appendix S8 **The number of nests predated followed by the number of nests monitored in each habitat classification and substrate type per study site.**
(DOCX)
