# Supplementary material for: Living with Aliens: Effects of Invasive Shrub Honeysuckles on Avian Nesting
Source: PLoS One. 2014 Sep 17;9(9):e107120. doi: 10.1371/journal.pone.0107120 (PMC4167549; doi:10.1371/journal.pone.0107120)
Supplement: Appendix S1 — Characteristics of the seven study sites. (DOCX) [file pone.0107120.s001.docx]

**Appendix S1:** Characteristics of the seven study sites. Included in the table are the size, dominant shrub cover, woody vegetation community, and abiotic characteristics.

| Site | Searched Area (ha) | Dominate Shrub Cover | Shrub Community | Canopy Community | Site Characteristics |
| --- | --- | --- | --- | --- | --- |
| Millbrook Marsh (Site 1) 40°48'48.19"N 77°50'04.77"W | 12.5 | HS | *Lonicera* spp*. Cornus amomum  Cornus racemosa Cornus seriea Viburnum* spp*. Malus* spp*. Rosa multiflora  Ligustrum* spp*.* | *Juglans nigra Acer negundo Quercus* spp*. Prunus serotina Salix* spp | - A wetland located in State College, PA.  - Shrub community covered most of the area - Heterogeneous canopy. |
|  |  |  |  |  |  |
| Lederer Park (Site 2) 40°47'47.66"N  77°50'26.39"W | 5.1 | HS | *Lonicera* spp*. Cornus amomum  Cornus racemosa Rhamnus carthartica  Ligustrum* spp*.* | *Carya* spp*. Populus* spp*. Pinus* spp*. Prunus serotina Gleditsia triacanthos* | - A forested area located in State College  - Almost completely covered by a canopy - Only site without a water source |
|  |  |  |  |  |  |
| Walnut Springs (Site 3) 40°48'09.47"N  77°50'19.90"W | 6.9 | HS | *Lonicera* spp*. Cornus amomum  Cornus racemosa Cornus seriea Crataegus* spp*. Rosa multiflora  Ligustrum* spp*.* | *Juglans nigra Quercus* spp. | - Located in State College  - Along a collection of springs - With two small wetland areas.  - Covered with a canopy |
|  |  |  |  |  |  |
| Shaver's Creek (Site 4) 40°40'04.94"N  77°54'59.29'W | 1.7 | HS | *Lonicera* spp*. Ellaeagnus umbellata  Cornus racemosa Crataegus* spp*. Rosa multiflora  Ligustrum* spp*.* | *Juglans nigra* | - Shrubland along a small stream In PSU’s Stone Valley forest - Approximately 20km from State College - Partially covered by a canopy |

**Appendix S1 (continued)**:

| Site | Searched Area (ha) | Dominate Shrub Cover | Shrub Community | Canopy Community | Site Characteristics |
| --- | --- | --- | --- | --- | --- |
| Woodcock Trailhead (Site 5) 40°40'36.33"N  77°54'23.50"W | 0.3 | NHS | *Lonicera* spp*. Frangula alnus Cornus amomum  Cornus racemosa Alnus incana Crataegus* spp*. Rosa multiflora  Ligustrum* spp*.* | *Acer* spp*. Quercus* spp. | - A wetland - In PSU’s Stone Valley forest - Approximately 20km from State College - Partially covered by a canopy |
|  |  |  |  |  |  |
| Boardwalk Trail (Site 6) 40°39'59.76"N  77°54'29.04"W | 0.4 | NHS | *Lonicera* spp*. Frangula alnus Cornus amomum  Cornus racemosa Cornus seriea Alnus incana Sambucus nigra Rosa multiflora* | *Pinus* spp*. Quercus* spp. | - Shrub habitat with two streams  - In PSU’s Stone Valley forest - Approximately 20km from State College - Small portion covered by a canopy |
|  |  |  |  |  |  |
| Masseyburg (Site 7) 40°38'40.55"N  77°55'52.74"W | 3.8 | NHS | *Carpinus caroliniana Cornus amomum Cornus racemosa Cornus seriea Crataegus* spp*. Ellaeagnus umbellata  Ilex verticillata Lindera benzoin Lonicera* spp*.  Ligustrum* spp*. Rosa multiflora* | *Carya* spp*. Pinus* spp*. Quercus* spp. | - An area of forest that runs along a creek  - In PSU’s Stone Valley forest - Approximately 20km from State College - Mostly covered by a canopy |
