# Supplementary material for: Living with Aliens: Effects of Invasive Shrub Honeysuckles on Avian Nesting
Source: PLoS One. 2014 Sep 17;9(9):e107120. doi: 10.1371/journal.pone.0107120 (PMC4167549; doi:10.1371/journal.pone.0107120)
Supplement: Appendix S2 — Average honeysuckle cover per habitat classification for each study site. (DOCX) [file pone.0107120.s002.docx]

## Appendix S2: Average honeysuckle cover per habitat classification for each study site. Averages were calculated from vegetation survey points and the vegetation data around the nests.

|  | Honeysuckle % Cover | | |
| --- | --- | --- | --- |
| Site | Low | Medium | High |
| Site 1 | 5.93 | 45.95 | 79.67 |
| Site 2 | 9.40 | 48.24 | 74.75 |
| Site 3 | 6.25 | 45.47 | 76.06 |
| Site 4 | 11.43 | 45.00 | 72.50 |
| Site 5 | 5.33 | NA* | 75.00 |
| Site 6 | 4.00 | 45.00 | 70.00 |
| Site 7 | 3.96 | 45.00 | 67.50 |

## * Site 5 had no data points points and nest vegetation data belonging to the medium habitat classification.
