# Supplementary material for: Living with Aliens: Effects of Invasive Shrub Honeysuckles on Avian Nesting
Source: PLoS One. 2014 Sep 17;9(9):e107120. doi: 10.1371/journal.pone.0107120 (PMC4167549; doi:10.1371/journal.pone.0107120)
Supplement: Appendix S3 — Number of observed nests built during the study year for the bird community. (DOCX) [file pone.0107120.s003.docx]

**Appendix S3:** Number of observed nests built during the study year for the bird community. Most nests (72.5%) belonged to *Dumetella carolinensis* and *Turdus Migratorius.*

| Species | Number nests |
| --- | --- |
| Northern Cardinal  (*Cardinalis cardinalis*) | 14 |
| Yellow Warbler  (*Setophaga petechia*) | 3 |
| Gray Catbird  (*Dumetella carolinensis*) | 112 |
| Acadian Flycatcher  (*Epidonax virescens*) | 1 |
| Batimore Oriole  (*Icterus galbula*) | 1 |
| Song Sparrow  (*Melospiza melodia*) | 3 |
| American Redstart  (*Setophaga ruticilla*) | 1 |
| Brown Thrasher  (*Toxostoma rufum*) | 1 |
| American Robin  (*Turdus Migratorius*) | 49 |
| Mourning Dove  (*Zenaida macroura*) | 1 |
| Unknown spp.* | 36 |
| Total | 222 |

*Unknown spp. nests were only used in the nest habitat and substrate usage analyses and not the predation analyses
