# Supplementary material for: Living with Aliens: Effects of Invasive Shrub Honeysuckles on Avian Nesting
Source: PLoS One. 2014 Sep 17;9(9):e107120. doi: 10.1371/journal.pone.0107120 (PMC4167549; doi:10.1371/journal.pone.0107120)
Supplement: Appendix S4 — Number of observed nests built during the study year in each habitat classification and substrate type per study site. (DOCX) [file pone.0107120.s004.docx]

## Appendix S4: Number of observed nests built during the study year in each habitat classification and substrate type per study site. Catbirds were the only species observed nesting in all of the sites.

| Site | Species | HS Level | | |  | Substrate | | Total number of nests |
| --- | --- | --- | --- | --- | --- | --- | --- | --- |
|  |  | High | Medium | Low |  | HS | NHS |  |
| Site 1 (12.5ha) | Gray Catbird (*Dumetella carolinensis*) | 23 | 15 | 10 |  | 38 | 10 | 48 |
|  | Northern Cardinal (*Cardinalis cardinalis*) | 1 | 0 | 2 |  | 3 | 1 | 4 |
|  | American Robin (*Turdus migratorius*) | 8 | 6 | 6 |  | 10 | 10 | 20 |
|  | Yellow Warbler (*Setophaga petechia*) | 1 | 0 | 2 |  | 1 | 2 | 3 |
|  | Song Sparrow (*Melospiza melodia*) | 1 | 0 | 2 |  | 1 | 2 | 3 |
|  | American Redstart (*Setophaga ruticilla*) | 0 | 0 | 1 |  | 0 | 1 | 1 |
|  | Batimore Oriole (*Icterus galbula*) | 0 | 0 | 1 |  | 0 | 1 | 1 |
|  | Mourning Dove (*Zenaida macroura*) | 0 | 0 | 1 |  | 1 | 0 | 1 |
|  | Unknown Species* | 7 | 5 | 9 |  | 12 | 9 | 21 |
|  | Total | 41 | 26 | 34 |  | 66 | 36 | 102 |

**Appendix S4 (continued):**

|  |  |  |  |  |  |  |  |  |
| --- | --- | --- | --- | --- | --- | --- | --- | --- |
| Site | Species | HS Level | | |  | Substrate | | Total number of nests |
|  |  | High | Medium | Low |  | HS | NHS |  |
| Site 2 (5.1ha) | Gray Catbird (*Dumetella carolinensis*) | 4 | 5 | 3 |  | 9 | 3 | 12 |
|  | Northern Cardinal (*Cardinalis cardinalis*) | 2 | 1 | 2 |  | 2 | 3 | 4 |
|  | American Robin (*Turdus migratorius*) | 1 | 5 | 2 |  | 3 | 5 | 8 |
|  | Unknown Species* | 1 | 0 | 1 |  | 0 | 2 | 2 |
|  | Total | 8 | 11 | 8 |  | 14 | 13 | 26 |
|  |  |  |  |  |  |  |  |  |
| Site | Species | HS Level | | |  | Substrate | | Total number of nests |
|  |  | High | Medium | Low |  | HS | NHS |  |
| Site 3 (6.9ha) | Gray Catbird (*Dumetella carolinensis*) | 5 | 2 | 2 |  | 7 | 2 | 9 |
|  | Northern Cardinal (*Cardinalis cardinalis*) | 1 | 2 | 1 |  | 3 | 1 | 4 |
|  | American Robin (*Turdus migratorius*) | 13 | 7 | 1 |  | 19 | 2 | 21 |
|  | Unknown Species* | 2 | 0 | 0 |  | 0 | 2 | 2 |
|  | Total | 21 | 11 | 4 |  | 29 | 7 | 36 |

**Appendix S4 (continued):**

|  |  |  |  |  |  |  |  |  |
| --- | --- | --- | --- | --- | --- | --- | --- | --- |
| Site | Species | HS Level | | |  | Substrate | | Total number of nests |
|  |  | High | Medium | Low |  | HS | NHS |  |
| Site 4 (1.7ha) | Gray Catbird (*Dumetella carolinensis*) | 2 | 3 | 2 |  | 6 | 1 | 7 |
|  | Northern Cardinal (*Cardinalis cardinalis*) | 0 | 0 | 2 |  | 0 | 2 | 2 |
|  | Unknown Species* | 1 | 1 | 2 |  | 3 | 1 | 4 |
|  | Total | 3 | 4 | 6 |  | 9 | 4 | 13 |
|  |  |  |  |  |  |  |  |  |
| Site | Species | HS Level | | |  | Substrate | | Total number of nests |
|  |  | High | Medium | Low |  | HS | NHS |  |
| Site 5 (0.3ha) | Gray Catbird (*Dumetella carolinensis*) | 0 | 0 | 11 |  | 2 | 9 | 11 |
|  | Unknown Species* | 0 | 0 | 1 |  | 0 | 1 | 1 |
|  | Total | 0 | 0 | 12 |  | 2 | 10 | 12 |
|  |  |  |  |  |  |  |  |  |
| Site | Species | HS Level | | |  | Substrate | | Total number of nests |
|  |  | High | Medium | Low |  | HS | NHS |  |
| Site 6 (0.4ha) | Gray Catbird (*Dumetella carolinensis*) | 6 | 2 | 0 |  | 2 | 6 | 8 |
|  | Unknown Species* | 1 | 3 | 0 |  | 2 | 2 | 4 |
|  | Total | 7 | 5 | 0 |  | 4 | 8 | 12 |

**Appendix S4 (continued):**

|  |  |  |  |  |  |  |  |  |
| --- | --- | --- | --- | --- | --- | --- | --- | --- |
| Site | Species | HS Level | | |  | Substrate | | Total number of nests |
|  |  | High | Medium | Low |  | HS | NHS |  |
| Site 7 (3.8ha) | Gray Catbird (*Dumetella carolinensis*) | 2 | 1 | 14 |  | 2 | 15 | 17 |
|  | Brown Thrasher (*Toxostoma rufum*) | 0 | 0 | 1 |  | 0 | 1 | 1 |
|  | Acadian Flycatcher (*Empidonax virescens*) | 0 | 0 | 1 |  | 0 | 1 | 1 |
|  | Unknown Species* | 0 | 0 | 2 |  | 1 | 1 | 2 |
|  | Total | 2 | 1 | 18 |  | 3 | 18 | 21 |

* Unknown spp. nests were only used in the nest habitat and substrate usage analyses and not the predation analyses.
