# Supplementary material for: Living with Aliens: Effects of Invasive Shrub Honeysuckles on Avian Nesting
Source: PLoS One. 2014 Sep 17;9(9):e107120. doi: 10.1371/journal.pone.0107120 (PMC4167549; doi:10.1371/journal.pone.0107120)
Supplement: Appendix S5 — Percent cover of plant species around nests. (DOCX) [file pone.0107120.s005.docx]

**Appendix S5:** Percent cover of plant species around nests. *Lonicera morrowii* was the most abundant followed by *Vitis* species.

| Species | Percent Cover |
| --- | --- |
| *Berberis* spp. ^†^ | 0.46 |
| *Celastrus orbiculatus*^†^ | 2.32 |
| *Cornus* *amomum* | 5.08 |
| *Cornus racemosa* | 4.07 |
| *Cornus sericea* | 0.81 |
| *Crataegus* spp.^†^ | 0.16 |
| *Elaeagnus umbellata*^†^ | 2.89 |
| *Ligustrum obtusifolium*^†^ | 5.93 |
| *Lonicera maackii*^†^ | 0.16 |
| *Lonicera morrowii*^†^ | 38.26 |
| *Malus* spp. | 0.78 |
| *Parthenocissus quinquefolia* | 1.24 |
| *Phytolacca americana* | 0.27 |
| *Prunus serotina* | 1.09 |
| *Prunus* spp. | 0.04 |
| *Rhamnus cathartica*^†^ | 0.85 |
| *Rosa multiflora*^†^ | 7.13 |
| *Rubus occidentalis* | 2.09 |
| *Sambucus nigra* | 0.70 |
| *Ulmus* spp. | 0.08 |
| *Vitis* spp*.* | 8.12 |
| Unknown species | 2.52 |

^†^Considered invasive by the USDA
