# Supplementary material for: Living with Aliens: Effects of Invasive Shrub Honeysuckles on Avian Nesting
Source: PLoS One. 2014 Sep 17;9(9):e107120. doi: 10.1371/journal.pone.0107120 (PMC4167549; doi:10.1371/journal.pone.0107120)
Supplement: Appendix S6 — The parental care data obtained from video recordings taken when the nestlings were 4–5 days old. (DOCX) [file pone.0107120.s006.docx]

**Appendix S6:** The parental care data obtained from video recordings taken when the nestlings were 4-5 days old. HSD stand for honeysuckle dominated habitats and NAT stand for native dominated habitats. GRCA stands for Gray Catbird (*Dumetella carolinensis*)

| Site | Habitat | Species | Nest ID | Num. of Visits | Time of Recording (min) | Total Time Present at Nest (min) | Num. of Feeding Visits | Num. of Visits of Unk. Activity | Num. of Visits with Invert. Food | Num. of Visits with Fruit Food | Num. of Visits with Unk. Food |
| --- | --- | --- | --- | --- | --- | --- | --- | --- | --- | --- | --- |
| Site 1 | HSD | GRCA | 4 | 185 | 528.52 | 140.84 | 185 | 0 | 32 | 0 | 153 |
| Site 1 | HSD | GRCA | 10 | 163 | 454.02 | 156.85 | 155 | 2 | 0 | 0 | 155 |
| Site 1 | HSD | GRCA | 51 | 141 | 464.73 | 159.40 | 141 | 0 | 94 | 35 | 15 |
| Site 1 | HSD | GRCA | 53 | 160 | 489.30 | 51.72 | 160 | 0 | 131 | 18 | 11 |
| Site 1 | HSD | GRCA | 69 | 136 | 504.80 | 224.12 | 133 | 0 | 90 | 30 | 13 |
| Site 1 | HSD | GRCA | 79 | 116 | 519.92 | 257.87 | 113 | 2 | 104 | 5 | 4 |
| Site 1 | HSD | GRCA | 109 | 149 | 524.98 | 230.98 | 143 | 1 | 50 | 20 | 73 |
| Site 1 | HSD | GRCA | 110 | 79 | 526.77 | 228.18 | 61 | 16 | 13 | 7 | 41 |
| Site 2 | HSD | GRCA | 15 | 99 | 502.09 | 258.15 | 95 | 1 | 69 | 0 | 26 |
| Site 2 | HSD | GRCA | 22 | 165 | 515.29 | 73.98 | 163 | 1 | 10 | 0 | 153 |
| Site 3 | HSD | GRCA | 130 | 128 | 523.20 | 250.83 | 123 | 5 | 41 | 11 | 71 |
| Site 3 | HSD | GRCA | 16 | 196 | 513.51 | 55.98 | 196 | 0 | 168 | 6 | 22 |
| Site 5 | NAT | GRCA | 32 | 153 | 527.22 | 172.53 | 151 | 0 | 141 | 0 | 10 |
| Site 5 | NAT | GRCA | 33 | 112 | 445.22 | 140.88 | 112 | 0 | 96 | 0 | 16 |
| Site 5 | NAT | GRCA | 92 | 83 | 422.24 | 169.57 | 73 | 0 | 55 | 5 | 14 |
| Site 5 | NAT | GRCA | 135 | 78 | 530.45 | 169.30 | 76 | 0 | 21 | 20 | 35 |
| Site 6 | NAT | GRCA | 103 | 70 | 506.97 | 296.07 | 66 | 1 | 65 | 0 | 1 |
| Site 6 | NAT | GRCA | 149 | 90 | 534.83 | 144.63 | 88 | 1 | 46 | 0 | 43 |
| Site 6 | NAT | GRCA | 55 | 103 | 503.53 | 204.00 | 101 | 0 | 91 | 1 | 9 |
| Site 7 | NAT | GRCA | 72 | 121 | 495.18 | 240.35 | 120 | 0 | 75 | 2 | 43 |
| Site 7 | NAT | GRCA | 136 | 88 | 551.07 | 202.87 | 86 | 2 | 42 | 9 | 35 |
| Site 7 | NAT | GRCA | 138 | 78 | 519.78 | 166.53 | 78 | 0 | 40 | 10 | 28 |
| Site 7 | NAT | GRCA | 25 | 87 | 421.17 | 150.35 | 85 | 2 | 0 | 0 | 85 |
