# Supplementary material for: Living with Aliens: Effects of Invasive Shrub Honeysuckles on Avian Nesting
Source: PLoS One. 2014 Sep 17;9(9):e107120. doi: 10.1371/journal.pone.0107120 (PMC4167549; doi:10.1371/journal.pone.0107120)
Supplement: Appendix S7 — The morphological measurements of nestlings at age 8–9 days. (DOCX) [file pone.0107120.s007.docx]

**Appendix S7:** The morphological measurements of nestlings at age 8-9 days. HSD stand for honeysuckle dominated habitats and NAT stand for native dominated habitats. GRCA stands for Gray Catbird (*Dumetella carolinensis*)

| Plot ID | Habitat | Species | Nest ID | Nestling | Mass (g) | Tarsus Length (mm) |
| --- | --- | --- | --- | --- | --- | --- |
| Site 1 | HSD | GRCA | 4 | 1 | 29.5 | 28.0 |
|  |  |  |  | 2 | 30.0 | 27.0 |
| Site 1 | HDS | GRCA | 10 | 1 | 27.0 | 27.0 |
|  |  |  |  | 2 | 28.0 | 27.0 |
| Site 1 | HSD | GRCA | 112 | 1 | 29.5 | 27.69 |
|  |  |  |  | 2 | 28.5 | 26.90 |
| Site 1 | HSD | GRCA | 51 | 1 | 29.0 | 26.59 |
|  |  |  |  | 2 | 31.5 | 25.52 |
| Site 1 | HSD | GRCA | 69 | 1 | 31.0 | 27.73 |
|  |  |  |  | 2 | 29.0 | 28.42 |
| Site 1 | HSD | GRCA | 110 | 1 | 29.0 | 26.72 |
|  |  |  |  | 2 | 28.0 | 27.89 |
| Site 2 | HSD | GRCA | 15 | 1 | 31.0 | 27.5 |
|  |  |  |  | 2 | 30.0 | 27.5 |
| Site 2 | HSD | GRCA | 22 | 1 | 30.0 | 28.15 |
|  |  |  |  | 2 | 31.0 | 27.81 |
| Site 2 | HSD | GRCA | 108 | 1 | 28.0 | 28.26 |
|  |  |  |  | 2 | 27.0 | 27.76 |
| Site 5 | NAT | GRCA | 135 | 1 | 27.0 | 25.76 |
|  |  |  |  | 2 | 30.0 | 27.09 |
| Site 5 | NAT | GRCA | 92 | 1 | 33.0 | 28.88 |
|  |  |  |  | 2 | 29.0 | 27.93 |
| Site 6 | NAT | GRCA | 103 | 1 | 28.0 | 25.82 |
|  |  |  |  | 2 | 30.0 | 26.38 |
| Site 6 | NAT | GRCA | 149 | 1 | 29.5 | 26.06 |
|  |  |  |  | 2 | 27.5 | 26.86 |
| Site 6 | NAT | GRCA | 109 | 1 | 27.0 | 27.84 |
|  |  |  |  | 2 | 29.0 | 28.51 |
