# Supplementary material for: Living with Aliens: Effects of Invasive Shrub Honeysuckles on Avian Nesting
Source: PLoS One. 2014 Sep 17;9(9):e107120. doi: 10.1371/journal.pone.0107120 (PMC4167549; doi:10.1371/journal.pone.0107120)
Supplement: Appendix S8 — The number of nests predated followed by the number of nests monitored in each habitat classification and substrate type per study site. (DOCX) [file pone.0107120.s008.docx]

**Appendix S8:** The number of nests predated followed by the number of nests monitored (in parenthesis) in each habitat classification and substrate type per study site is shown.

-Nests predated (nests monitored)

| Site | Species | HS Level | | |  | Substrate | | Total nests predated |
| --- | --- | --- | --- | --- | --- | --- | --- | --- |
|  |  | High | Medium | Low |  | HS | NHS |  |
| Site 1 (12.5ha) | Gray Catbird (*Dumetella carolinensis*) | 5(17) | 1(7) | 2(6) |  | 6(23) | 2(7) | 8(30) |
|  | Northern Cardinal (*Cardinalis cardinalis*) | 0(1) | 1(1) | 0(2) |  | 1(2) | 0(2) | 1(4) |
|  | American Robin (*Turdus migratorius*) | 1(1) | 2(3) | 0(2) |  | 3(3) | 0(3) | 3(6) |
|  | Yellow Warbler (*Setophaga petechia*) | 0(1) | 0(0) | 1(2) |  | 0(1) | 1(2) | 1(3) |
|  | Song Sparrow (*Melospiza melodia*) | 0(1) | 0(1) | 0(1) |  | 0(1) | 0(2) | 0(3) |
|  | American Redstart (*Setophaga ruticilla*) | 0(0) | 0(0) | 0(1) |  | 0(0) | 0(1) | 0(1) |
|  | Batimore Oriole (*Icterus galbula*) | 0(0) | 0(0) | 0(1) |  | 0(0) | 0(1) | 0(1) |
|  | Mourning Dove (*Zenaida macroura*) | 0(0) | 0(0) | 0(1) |  | 0(0) | 0(1) | 0(1) |
|  | Total | 6(21) | 4(12) | 3(16) |  | 10(30) | 3(19) | 13(49) |

**Appendix S8 (continued):**

|  |  |  |  |  |  |  |  |  |
| --- | --- | --- | --- | --- | --- | --- | --- | --- |
| Site | Species | HS Level | | |  | Substrate | | Total nests predated |
|  |  | High | Medium | Low |  | HS | NHS |  |
| Site 2 (5.1ha) | Gray Catbird (*Dumetella carolinensis*) | 1(4) | 0(3) | 1(2) |  | 1(7) | 1(2) | 2(9) |
|  | Northern Cardinal (*Cardinalis cardinalis*) | 1(1) | 0(1) | 1(2) |  | 1(2) | 1(2) | 2(4) |
|  | American Robin (*Turdus migratorius*) | 0(0) | 0(2) | 0(1) |  | 0(1) | 0(2) | 0(3) |
|  | Total | 2(5) | 0(6) | 2(5) |  | 2(10) | 2(6) | 4(16) |
|  |  |  |  |  |  |  |  |  |
| Site | Species | HS Level | | |  | Substrate | | Total nests predated |
|  |  | High | Medium | Low |  | HS | NHS |  |
| Site 3 (6.9ha) | Gray Catbird (*Dumetella carolinensis*) | 1(5) | 2(2) | 0(2) |  | 2(7) | 1(2) | 3(9) |
|  | Northern Cardinal (*Cardinalis cardinalis*) | 0(1) | 1(2) | 0(1) |  | 1(3) | 0(1) | 1(4) |
|  | American Robin (*Turdus migratorius*) | 2(9) | 2(6) | 0(1) |  | 4(14) | 0(2) | 4(16) |
|  | Total | 3(15) | 6(10) | 0(4) |  | 7(24) | 1(5) | 8(29) |
|  |  |  |  |  |  |  |  |  |

**Appendix S8 (continued):**

|  |  |  |  |  |  |  |  |  |
| --- | --- | --- | --- | --- | --- | --- | --- | --- |
| Site | Species | HS Level | | |  | Substrate | | Total nests predated |
|  |  | High | Medium | Low |  | HS | NHS |  |
| Site 5 (0.3ha) | Gray Catbird (*Dumetella carolinensis*) | 0(0) | 0(0) | 4(9) |  | 0(1) | 4(8) | 4(9) |
|  | Total | 0(0) | 0(0) | 4(9) |  | 0(1) | 4(8) | 4(9) |
|  |  |  |  |  |  |  |  |  |
| Site | Species | HS Level | | |  | Substrate | | Total nests predated |
|  |  | High | Medium | Low |  | HS | NHS |  |
| Site 6 (0.4ha) | Gray Catbird (*Dumetella carolinensis*) | 0(0) | 1(2) | 2(6) |  | 1(2) | 2(6) | 3(10) |
|  | Total | 0(0) | 1(2) | 2(6) |  | 1(2) | 2(6) | 3(10) |
|  |  |  |  |  |  |  |  |  |
| Site | Species | HS Level | | |  | Substrate | | Total nests predated |
|  |  | High | Medium | Low |  | HS | NHS |  |
| Site 7 (3.8ha) | Gray Catbird (*Dumetella carolinensis*) | 2(2) | 1(1) | 1(10) |  | 2(2) | 2(11) | 4(13) |
|  | Brown Thrasher (*Toxostoma rufum*) | 0(0) | 0(0) | 0(1) |  | 0(0) | 0(1) | 0(1) |
|  | Acadian Flycatcher (*Empidonax virescens*) | 0(0) | 0(0) | 0(1) |  | 0(0) | 0(1) | 0(1) |
|  | Total | 2(2) | 1(1) | 1(12) |  | 2(2) | 2(13) | 4(15) |
